# Left Ventricular Failure Despite Patent Coronary Arteries in Acute Type A Aortic Dissection

**DOI:** 10.1093/icvts/ivag204

**Published:** 2026-07-21

**Authors:** Daisuke Nakatsuka, Shun Sato, Yuichi Tara, Yasumasa Tsukamoto

**Affiliations:** Department of Cardiovascular Surgery, Nara Prefecture General Medical Center, Nara 630-8581, Japan; Department of Cardiovascular Surgery, Nara Prefecture General Medical Center, Nara 630-8581, Japan; Department of Cardiovascular Surgery, Nara Prefecture General Medical Center, Nara 630-8581, Japan; Department of Cardiovascular Surgery, National Cerebral and Cardiovascular Center, Suita 564-8565, Japan

**Keywords:** acute type A aortic dissection, left ventricular failure, mechanical circulatory support, left ventricular assist device

## Abstract

Severe myocardial failure after acute type A aortic dissection is usually attributed to structural coronary malperfusion. We report catastrophic global left ventricular failure despite preserved epicardial coronary anatomy, successfully bridged with durable mechanical circulatory support. A 57-year-old man presented with acute type A dissection and cardiogenic shock with extensive precordial ST-segment elevation. Emergency total arch replacement with a frozen elephant trunk was performed. Intraoperative inspection and postoperative coronary angiography confirmed patent coronary ostia and arteries. Postoperatively, venoarterial extracorporeal membrane oxygenation and a percutaneous microaxial flow pump was instituted; extracorporeal membrane oxygenation was weaned on day 4, but the patient remained pump-dependent. Perfusion imaging showed extensive fixed defects in the left coronary distribution, and durable left ventricular assist device implantation was required 6 weeks later. Severe myocardial injury can occur in acute type A aortic dissection without demonstrable fixed coronary obstruction. When dysfunction persists after successful repair, early viability assessment is essential to guide timely transition to durable mechanical circulatory support.

## INTRODUCTION

Myocardial ischemia complicating acute Stanford type A aortic dissection typically results from structural coronary malperfusion caused by intimal flap extension into the coronary ostia or true-lumen compression.^1^ Rarely, profound left ventricular failure occurs despite patent coronary arteries, which complicates diagnosis and decision-making regarding mechanical circulatory support. We report a patient with acute type A aortic dissection who developed irreversible global left ventricular failure in the absence of epicardial coronary obstruction and ultimately required durable left ventricular assist device implantation.

## CASE DESCRIPTION

**Figure 1.**
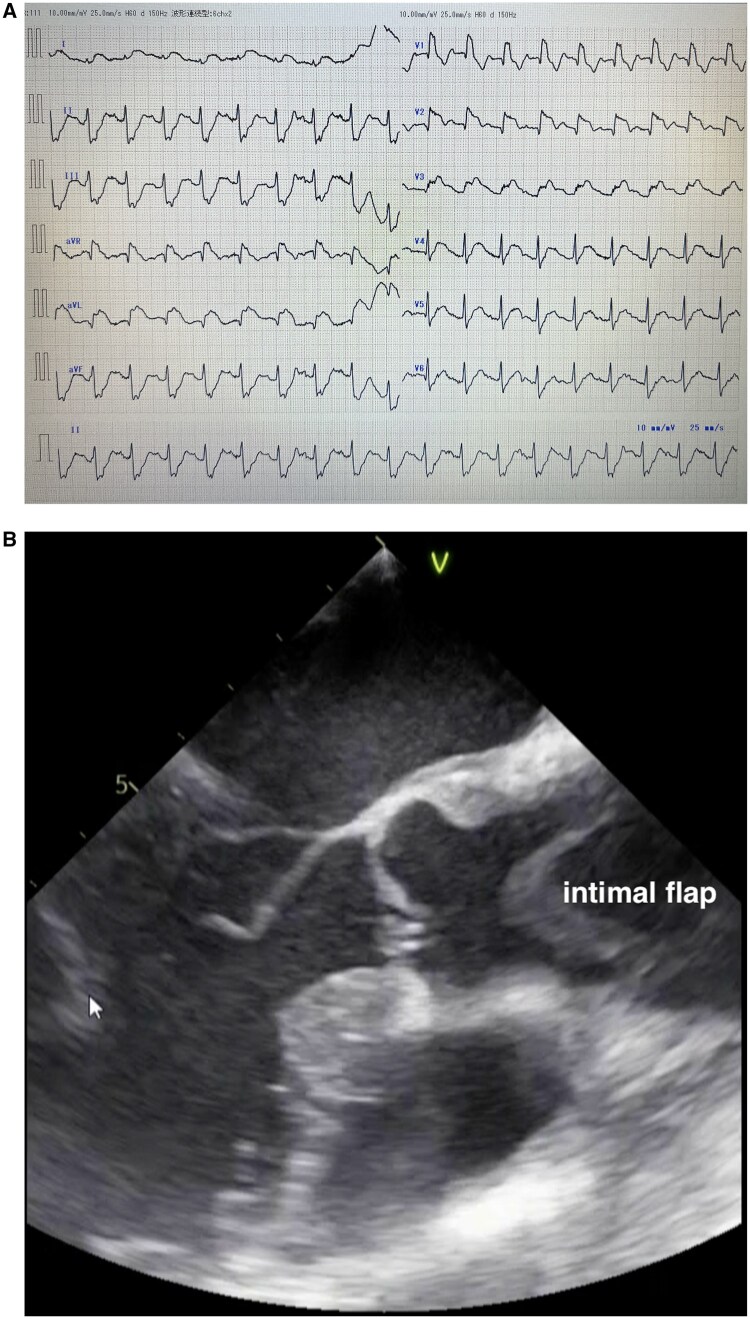
Preoperative Findings. (A) Twelve-lead electrocardiography showing extensive precordial ST-segment and T-wave elevation. (B) Transoesophageal echocardiography (mid-oesophageal long-axis view) showing proximal flap extension to the sinotubular junction without coronary ostial involvement.

**Figure 2.**
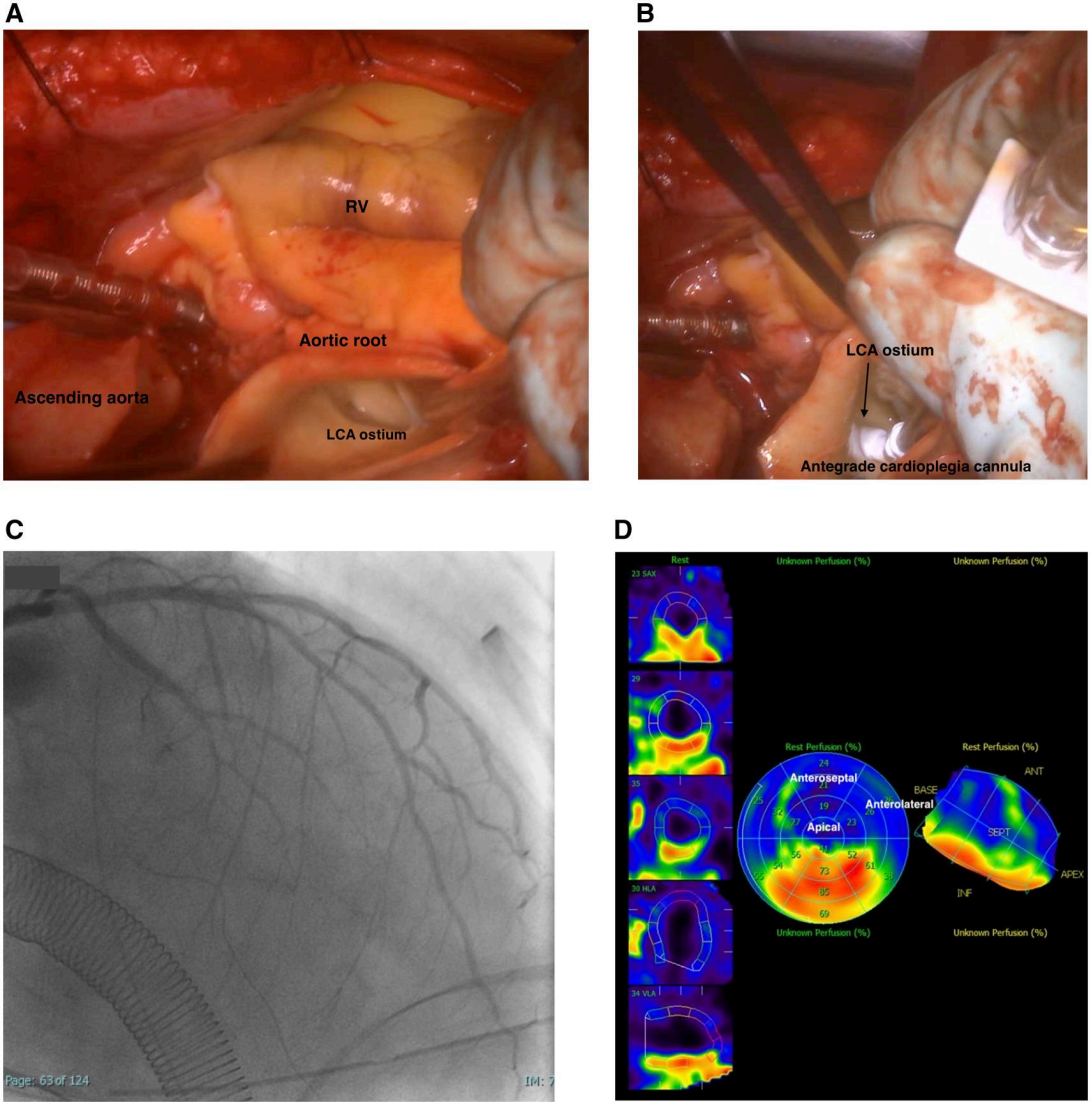
Intraoperative and Postoperative Assessment. (A) Intact left coronary ostium. (B) Selective antegrade cardioplegia into the left coronary ostium; the left coronary ostium and the antegrade cardioplegia cannula are labelled. (C) Postoperative coronary angiography showing patent coronary arteries. (D) Resting perfusion single-photon emission computed tomography showing extensive fixed defects in the left coronary distribution.

A 57-year-old man presented with acute Stanford type A aortic dissection and cardiogenic shock. His medical history included untreated hypertension and active smoking, with no known dyslipidaemia, diabetes mellitus, myocarditis, prior coronary artery disease, or documented preexisting left ventricular dysfunction, and no pre-admission cardiac medications. There was no pericardial tamponade on admission. Admission laboratory values were high-sensitivity troponin T 0.091 ng/mL, lactate 6.6 mmol/L, creatinine 1.36 mg/dL, aspartate aminotransferase 34 U/L and alanine aminotransferase 13 U/L. There was no cerebral malperfusion; coronary and right lower-limb malperfusion were present clinically. The dissection was classified as TEM type A, E1, M1(+) and M3(+), and Penn class Abc. Preoperative electrocardiography showed extensive precordial ST-segment elevation (**Figure 1A**). Computed tomography showed no coronary ostial involvement. Transoesophageal echocardiography demonstrated severe global left ventricular dysfunction (visual ejection fraction ∼20%); the intimal flap extended to the sinotubular junction without ostial involvement (**Figure 1B**). Creatine kinase/creatine kinase-MB rose from 219/19 U/L on arrival to 18 619/1396 U/L immediately postoperatively (∼11 h after symptom onset), with 16 764/398 U/L the next morning (creatine kinase-MB upper limit of normal <13 U/L), consistent with substantial myocardial necrosis. Emergency total arch replacement with a frozen elephant trunk was performed. Frozen elephant trunk repair was chosen for persistent false-lumen perfusion beyond the arch with descending true-lumen narrowing and reduced lower-limb perfusion, rather than for a discrete additional distal entry. Cannulation was via the right common femoral artery. The interval from symptom onset to cross-clamping was 4 h 30 min; cardiopulmonary bypass, cross-clamp, circulatory arrest, and retrograde and selective cerebral perfusion times were 4 h 56 min, 2 h 26 min, 59 min, 3 min and 1 h 54 min, respectively. Myocardial protection comprised initial retrograde crystalloid cardioplegia, then selective antegrade and intermittent retrograde dosing, with terminal antegrade and retrograde hot-shot cardioplegia. The coronary ostia were intact; selective antegrade cardioplegia was infused directly into the left coronary ostium (**Figure 2A and B**). Postoperative coronary angiography confirmed patent coronary arteries (**Figure 2C**). Postoperatively, venoarterial extracorporeal membrane oxygenation and a percutaneous microaxial flow pump (Impella 5.5; Abiomed) were initiated. Extracorporeal membrane oxygenation was weaned on postoperative day 4, but left ventricular function did not recover and the patient remained pump-dependent. Myocardial perfusion single-photon emission computed tomography demonstrated extensive fixed defects in the left coronary distribution (**Figure 2D**). He was transferred to an advanced heart failure centre, and durable left ventricular assist device implantation was performed 6 weeks after the index operation.

## DISCUSSION

Myocardial dysfunction in acute type A aortic dissection usually reflects structural coronary malperfusion.^1^ In the present case, however, direct intraoperative inspection, selective antegrade cardioplegia, and postoperative coronary angiography showed preserved epicardial coronary anatomy, whereas the electrocardiographic pattern, marked creatine kinase-MB elevation, and fixed perfusion defects indicated extensive myocardial necrosis. Severe myocardial injury can therefore occur even without demonstrable fixed coronary obstruction.

Although the presentation strongly suggested acute coronary ischemia, the precise mechanism could not be established retrospectively. The overall picture is most consistent with multifactorial non-obstructive coronary malperfusion, including severe low-flow ischemia in cardiogenic shock, transient dynamic ostial obstruction, vasomotor dysfunction, and microvascular failure, rather than isolated vasospasm alone.^2^ Takotsubo syndrome was considered less likely because echocardiography showed near-complete global akinesis rather than ballooning, and myocardial perfusion imaging demonstrated extensive fixed defects without viability. Concomitant coronary artery bypass grafting was not performed because no fixed lesion or suitable distal target was evident, and additional grafting would have prolonged myocardial ischemic time in an unstable patient. In the absence of a demonstrable additional distal entry, a simpler proximal strategy such as supracoronary ascending replacement would have been a reasonable alternative; frozen elephant trunk was nonetheless selected because of persistent distal false-lumen perfusion and lower-limb malperfusion. This more extensive repair increased operative complexity and may have prolonged the myocardial ischemic time, contributing to the irreversible injury. The mechanism is therefore best regarded as multifactorial, reflecting the preoperative shock state and possible non-obstructive coronary malperfusion compounded by prolonged intraoperative ischemia.

Combined extracorporeal membrane oxygenation and microaxial pump support provided haemodynamic stabilization and left ventricular unloading.^3^^,^^4^ When severe dysfunction persists after successful proximal repair despite patent coronary arteries, early viability assessment and timely referral to an advanced heart failure centre are essential to guide transition to durable mechanical circulatory support.^5^

## Data Availability

The data underlying this article will be shared on reasonable request to the corresponding author.
